# A Novel Microsurgical Robot: Preliminary Feasibility Test in Ophthalmic Field

**DOI:** 10.1167/tvst.11.8.13

**Published:** 2022-08-17

**Authors:** Alfonso Savastano, Stanislao Rizzo

**Affiliations:** 1 Ophthalmology Unit, Fondazione Policlinico Universitario A. Gemelli IRCCS, Rome, Italy; 2 Ophthalmology Unit, Università Cattolica del Sacro Cuore, Rome, Italy; 3 Consiglio Nazionale delle Ricerche, Istituto di Neuroscienze, Pisa, Italy

**Keywords:** robotic ophthalmic surgery, personalized medicine, robotic corneal graft, preliminary feasibility test, robotics

## Abstract

**Purpose:**

This study investigated the feasibility and potential advantage of using a new microsurgical teleoperated robot, Symani Surgical System, in the ophthalmology field. In particular, considering the extreme precision of the system and the dexterity of the instrument, possible use of the Symani Surgical System has been explored for suturing in corneal graft surgery.

**Methods:**

Manual and robot-assisted suturing of partial corneal transplants was performed on the porcine eye model by an ophthalmologist with experience with the porcine model. Suturing execution time, suture placement, and tomographic parameters were analyzed to assess the regularity and distribution of corneal sutures for both manual and robotic treatment.

**Results:**

The two robot-assisted procedures were properly completed on the porcine model, confirming the ease of use of the system and its capabilities, as well as the dexterity of the microinstruments. Manual and robotic treatments were found to be equivalent in terms of distance and angular precision of suture placement and corneal surface regularity (Gaussian anterior curvature). The robotic procedure required longer suturing execution times compared with the manual procedure.

**Conclusions:**

The technical and clinical feasibility of robot-assisted suturing of partial corneal graft using the Symani Surgical System has been confirmed for the first time, to our knowledge, using an ex vivo porcine model. Robotic suturing required longer time to complete but was equivalent to the manual procedure with regard to the imaging data collected.

**Translational Relevance:**

This study evaluated the use of the Symani Surgical System in the ophthalmology field. Future investigations could further identify the advantages offered by the stability, dexterity, and motion precision of the system for corneal surgeries, paving the way for clinical use in both adult and even more challenging pediatric therapy.

## Introduction

Current technology utilizing laser scanners and microscopes allows us to monitor retinal diseases at the microscopic level, but the anatomy and details that such systems visualize are beyond the physiological limit of what the human hand can operate on. Robotic systems open up a whole new chapter of eye surgery that was not possible before. Although robots have been developed for large-scale surgery, such as in the abdomen, until now only a few devices have been available that achieve the three-dimensional precision required to operate inside the human eye.^1^^–^^3^

Robotic surgery includes all types of surgical procedures that are aided by the use of a robotic system. Robotic-assisted surgery was developed to try to overcome the limitations of pre-existing minimally invasive and endoscopic surgical procedures and to enhance the capabilities of surgeons performing open surgery.

For robotic-assisted minimally invasive surgery, rather than manually moving the instruments, surgeons control the robotic instruments through the use of either a direct telemanipulator or computer control.

A telemanipulator is a remote console with manipulators that allow the surgeon to perform the movements associated with the surgery while robotic tools replicate those movements on the patient using dedicated end-effectors. In computer-controlled systems, the surgeon uses a computer to control the robotic arms and its end-effectors, although such systems can also use telemanipulators for their input. One advantage of the computerized method is that the surgeon does not have to be present, at least theoretically, leading to the possibility for remote surgery.

Robotic surgery has been criticized for its expense (e.g., an estimated increase of $1500 to $2000 per prostate surgery patient^4^), and there are still some concerns that it may not improve clinical outcomes in certain types of surgical procedures such as the prevention or treatment of breast cancer.^5^ However, the global market for surgical robots continues to grow at a compound annual growth rate of 10.4%, from $3.9 billion in 2018 to $6.5 billion by 2023.^6^

The Intuitive Surgical da Vinci surgical system is the market leader, but other options are emerging, including products designed for specific procedures that offer encouraging clinical outcomes.

The University of Oxford conducted a trial for the PRECEYES Surgical System. The test involved 12 patients who needed epiretinal membranes removed from their eyes or had a buildup of blood underneath the retina due to age-related macular degeneration. Half of the people underwent conventional procedures, and the others underwent robotic surgery. All of the surgeries were successful, but the robotic approach was at least as effective, and sometimes even more, than the procedures done manually.

The Intraocular Robotic Interventional Surgical System is a combined effort between the UCLA Jules Stein Eye Institute and the UCLA Department of Mechanical and Aerospace Engineering to provide a platform for performing complete ophthalmic procedures.

The Johns Hopkins Steady-Hand Eye Robot is a surgeon-initiated robot that is designed to share the control of surgical instruments with the retinal surgeon while filtering human hand tremor.^7^

Here, we describe the first use in ophthalmology field of a new robotically assisted system designed for microsurgery, the Symani Surgical System developed by Medical Microinstruments (Pisa, Italy).^8^ This robotic device was designed to drive one of the world's smallest wristed surgical instruments (NanoWrist microinstruments; MMI S.p.A, Pisa, Italy; U.S. Patent US10864051B2),^9^ with a wrist outer diameter (OD) of 3.5 mm, for the manipulation of millimeter and submillimeter soft tissues. In particular, the purpose of this feasibility study was to evaluate the performance of the Symani Surgical System on suturing of the partial thickness manual trephination of a porcine eye model compared with the traditional manual technique.

## Materials and Methods

### The Robotic System

**Figure 1.**
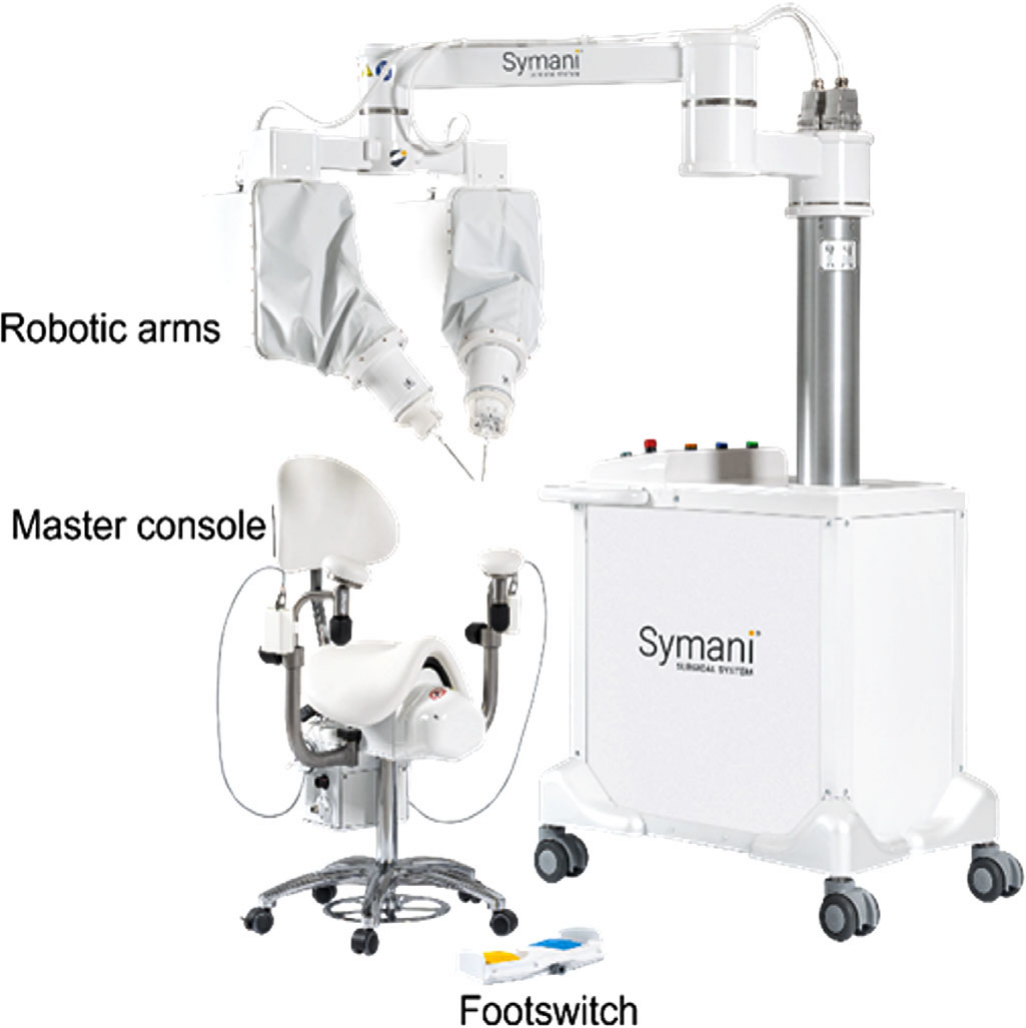
Overview of the Symani Surgical System.

The Symani Surgical System (Fig. 1) was designed to enhance reconstructive and microsurgery applications, and it received CE marking in 2019.^10^^,^^11^

The system consists of three main components^8^: cart with macropositioner and micromanipulators (CMM), console, and wristed miniaturized instruments. The CMM is the operative component of the Symani Surgical System that supports the robotic instruments on the surgical target. The micromanipulators are two robotic arms that manage the miniaturized instruments, allowing precise movement and providing seven degrees of freedom to the associated instrument (*x*, *y*, *z* movements; roll; pitch; yaw; and grip). The macropositioner is a mechanical arm where the micromanipulators are connected that allows the instruments to be placed precisely on the surgical site. The console is the control center for the Symani Surgical System, where the surgeon sits and handles the robotic instruments by means of two master controllers and a footswitch with two pedals. In real time, the hand movements are scaled (range, 7×–20×) and translated into the movement of the robotic instruments, thereby reducing tremor. The robotic instruments extend the surgeon's natural dexterity and range of motion compared with those of unaided human hands, allowing greater precision when operating in a microsurgical environment. The equipment required for standard suturing includes a set of robotic microinstruments such as a Micro Dilator, a Micro Needle Holder, and Micro Needle Holder Suture Cut (including a blade devoted to cutting the suture).

The Symani Surgical System does not incorporate any dedicated optical magnification system, because it was designed to be compatible with most current microsurgical microscopes.

In this study, the system was combined with the VITOM 3D magnification system (Karl Storz SE & Co. KG, Tuttlingen, Germany) and included two interconnected screens, one for the surgeon and the other one for the assistant.

In this setup, the surgeon simulated the surgical procedures while sitting at the console of the Symani Surgical System positioned on the side of the operating table and looking at a three-dimensional (3D) monitor with proper 3D glasses.

**Figure 2.**
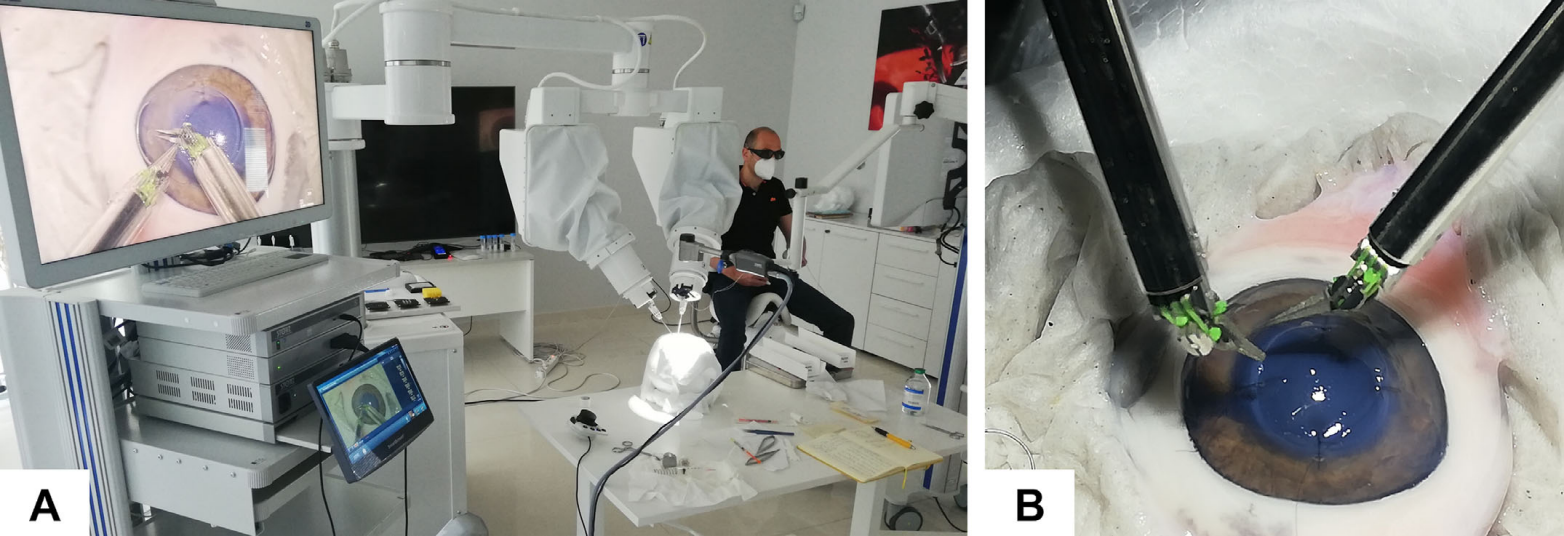
(A) Layout of the surgery simulation setup, and (B) detail of the robotic microinstrument performing stitches on a porcine corneal model.

The assisting surgeon was located beside the operating table, near the head simulator, to prepare and cut sutures with conventional manual microsurgery instruments or irrigate the surgical site. The surgeon assistant looked at a second 3D monitor positioned on the opposite side of the operating table (Fig. 2).

### Ex Vivo Corneal Simulators

The porcine eye is one of the most common simulators for clinical practice training in ophthalmic applications.^12^^,^^13^ The ex vivo porcine eyes were harvested with the entire eyeballs. Each eye was placed in a foam human head simulator fixed on an operating table.

### Surgical Procedure Simulation

An expert surgeon, with more than 10 years of experience in ophthalmology surgery and practice with ex vivo porcine models, performed three procedures utilizing the magnification of a VITOM 3D system. The first one was performed manually, and the other two were performed using the Symani Surgical System for the suturing phase after a preliminary 4-hour training session (Movie S1).

The eye surface was prepared by removing the external epithelial layer with the aid of tweezers. The human simulator foam head with the eyeball was then positioned on the operating table. A blue dermographic pen (GIMA S.p.A., Lombardy, Italy) was used to create a point of reference on the sclera of the porcine eye (12 o'clock). This point was then aligned with a corresponding point on the foam head in order to maintain the same intra- and postoperative orientation of the porcine eye. When the anterior chamber of the porcine eye was fixed with a clamp on a support, placed in front of the device, the clamp was rotated in order to align the reference point at 12 o'clock using the placido discs of the MS-39 device. Then, all of the necessary images of the anterior chamber were collected.

The cornea was trephined using a Barron vacuum corneal trephine (Katena Products, Parsippany-Troy Hills, NJ) to create a circular, deep, partial cut 9.0 mm in diameter. The trephination was about 800 µm in depth but was not complete, because the porcine cornea is thicker than the human one in the peripheral zone (about 1500 µm). The interrupted stitches technique was selected to repair the partial trephination, as it is one of the most widely used in clinical practice, depending on the status of the cornea assessed at the time of surgery.^15^ During each procedure, the surgeon placed 16 stitches with 10-0 interrupted sutures (DM133 monofilament nonabsorbable suture with spatula needle, 3/8 curvature, 6.6-mm chord; S&T AG, Neuhausen am Rheinfall, Switzerland) and a symmetrical arrangement.

During the robotic procedures, robotic arms with microinstruments were placed on either side of the head simulator with respect to the axis created by the midline position of the microscope. The tweezers were held in the non-dominant hand; the surgeon decided to use a Micro Dilator in the first robotic procedure but a Micro Needle Holder in the second one to improve tissue grip. With the dominant hand, the surgeon always used a Micro Needle Holder Suture Cut, a robotic instrument that includes blades in the proximal part of instrument tips devoted to suture cutting functionality, thus allowing the surgeon to replicate the standard procedure and setup where suture cutting is not required by the assistant.

### Data Collection and Quantitative Parameters

Data collection was carried out at three different time points.

#### Preoperative Phase

The corneal status was checked by using MS-39 anterior segment optical coherence tomography (AS-OCT; CSO Italy, Tuscany, Italy). The anterior chamber of the porcine eye was fixed with a clamp on a support and placed in front of the device, and preoperative tomographic images and sections were acquired.

Three tomographic images were acquired for each porcine eye. The best scan was selected for each case and, after recreating the best-fit cornea reconstruction allowed using the integrated software Phoenix 4 (CSO Italy), data about corneal surface curvature were extrapolated, considering the following parameters:

• Root mean square per area (RMS/A, µm/mm^2^), which represents the deviation of the surface examined from the aspherotoric best-fit surface characterized by four different parameters. The higher the RMS, the more irregular the corneal surface in the area delimited by the given diameter. Values for the anterior corneal surface were examined in correspondence to the available different corneal radii (3.0, 5.0, and 7.0 mm), and these values were averaged across radii.
• Gaussian anterior curvature (GAC, mm), which allows defining steep or flat curvature zones. The Gaussian curvature of a point on a surface is the “real” curvature (the geometric mean of the minimum and maximum curvature) and is independent of the direction of curvature measurement. For data extraction, each GAC map can be described as a set of concentric circumferences spaced out 0.2 mm between radii ranging from 0.0 to 6.0 mm. Available datasets contain 256 values of curvature for each ideal circumference. Data were then manually extracted from GAC maps corresponding to the 1.0-, 2.0-, 3.0-, and 4.0-mm corneal radii. This was the region selected as the most important one to achieve clear vision. The angular positions of the 16 different stitches were calculated and used to obtain data from preoperative images in correspondence with sutured regions. Intervals of five consecutive values of curvature were used for mean calculation corresponding to each interrupted stitch.

#### Intraoperative Phase

The simulation was documented with pictures and video recordings from the optical system. For each procedure, duration and successful completion of the surgery, as well as any unexpected events, were assessed in real time. Recordings were examined, and the different phases of each suture execution were timed using a digital timer. All times were rounded up to the nearest second.

The ensuing quantitative parameters were extracted:

• Surgery execution time—total time required to execute the surgical suturing procedure (placement of 16 interrupted stitches)
• Suture execution time—total time required to execute one single interrupted stitch

#### Postoperative Phase

Each operated eye, still situated in the foam head, was observed through a Dino Lite digital microscope (AM73915MZT; Dino-Lite Europe, Naarden, North Holland, the Netherlands), and images of eye surfaces with sutures were acquired at 20× magnification. Artificial tears (Thealoz Du; Théa Pharmaceuticals, Newcastle-under-Lyme, UK) were used to maintain wet and smooth corneal surfaces, which aided in collecting better quality images.

Digital images of sutured corneas were analyzed using DinoCapture 2.0 software (Dino-Lite Europe), and the following quantitative parameters were measured as indexes of the surgery precision:

• Distance precision (mm)—The distance between the entry for the needle and the intermediate corneal cut was measured directly on images, and the same was done for the exit point. The difference between the two distances was calculated; the smaller the value, the more precise was the corresponding stitch placement.
• Angular precision (°)—The angular displacement between each suture and the subsequent one was derived from angular measurements on pictures; the more homogeneous the displacements, the more precise was the circular distribution of the 16 stitches.

Corneal status was checked again by the MS39 device as described above, and postoperative tomographic images and sections were acquired. Images were analyzed to extract quantitative parameters as already discussed for the preoperative phase.

### Data Analysis

Surgery simulation execution time was recorded for each surgical treatment (the manual one and the first and second robotic ones), and individual values, mean values, and value distributions were calculated for each surgical treatment and compared among treatment types. Placement of sutures and corneal surface appearance were examined from a clinical point of view. With regard to the distance precision (mm) and angular precision (°) parameters, mean values and values distribution were calculated for each surgical treatment.

For each procedure, both preoperatively and postoperatively, RMS/A (µm/mm^2^) mean values across the different corneal radii (3.0, 5.0, and 7.0 mm) and value distributions were evaluated. GAC (mm) values were extracted from both preoperative and postoperative tomographic maps corresponding to sutured regions in order to make comparisons between the two different time points and different surgical treatments. Mean values were calculated first across sutures and then across the different corneal radii. Furthermore, only postoperative GAC data were examined across the 16 sutures and plotted for the different corneal radii.

Data analyses were performed using Prism 9.0 (GraphPad Software, San Diego, CA). For all of the above-mentioned parameters, quantitative comparisons were analyzed by using a two-way ANOVA test (repeated-measures, Sidak's multiple comparison test with 95% confidence intervals); *P* < 0.05 was considered significant.

## Results

The feasibility of suturing in a simulated partial corneal graft using a robotic approach combining the Symani Surgical System and VITOM 3D was confirmed. Two robot-assisted procedures were successfully completed on ex vivo porcine eyes, demonstrating superior motion precision and dexterity, together with an easy setup and procedure, due to use of the Needle Holder. No intraoperative complications or unexpected events were observed by the surgeon.

Quantitative evaluations supporting these claims are reported below.

**Figure 3.**
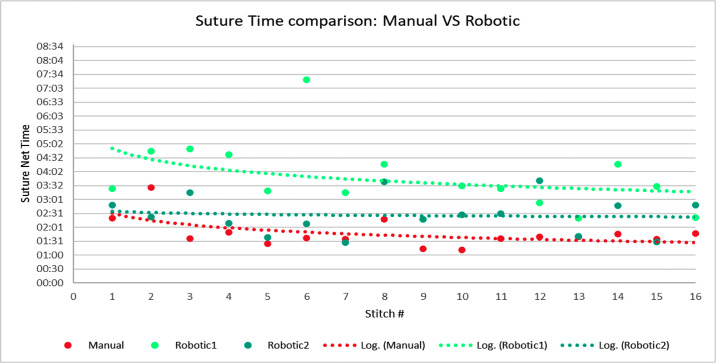
Single suture net time summary. The graph includes the execution net time for each single suture for all the three surgeries: manual (*red*), first robotic (*light green*), and second robotic (*dark green*).

**Figure 4.**
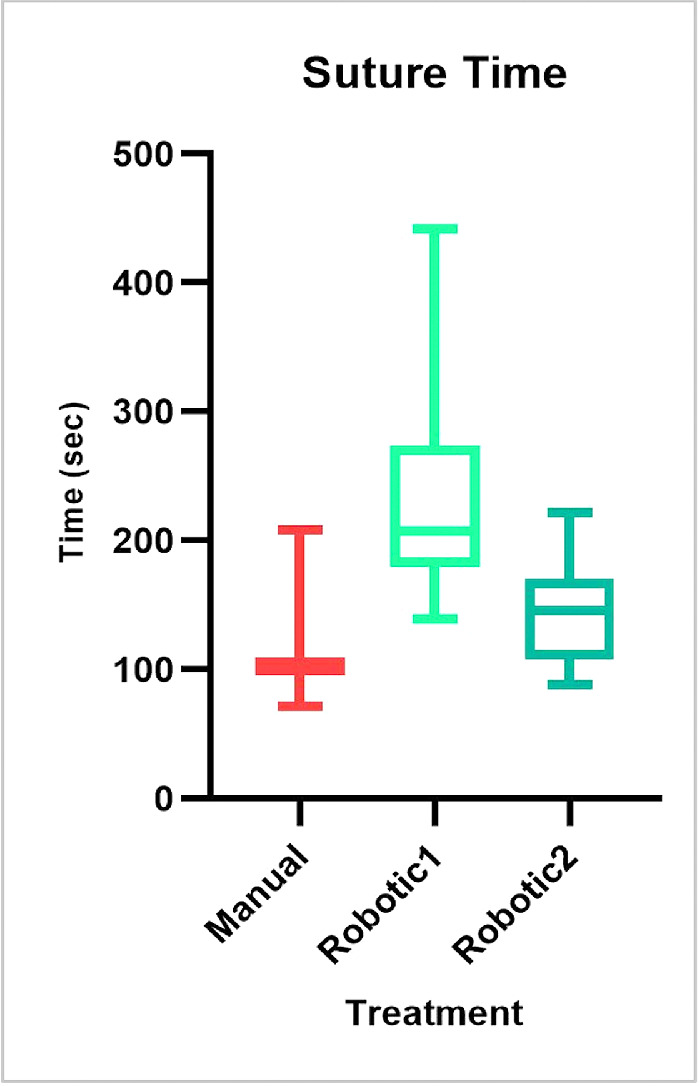
Mean suturing time and the distribution of values for each treatment.

The manual surgery lasted 28 minutes and 46 seconds; the first robotic treatment lasted 1 hour and 39 seconds, and the second robotic lasted 39 minutes and 24 seconds, highlighting the fast learning curve for use of the robotic system. A detailed analysis of one interrupted suture procedure indicated a similar trend. Figure 3 shows the suture time values for each stitch and each treatment; logarithmic trend lines were added to aid data visualization and to highlight possible learning mechanisms. Figure 4 displays mean suture execution times and the distribution of values; the ANOVA test showed that differences among mean values were statistically significant (*P* < 0.01) when comparing data for treatment. As expected, both of the robotic treatments had longer execution times with respect to the manual procedure, but the surgeon had not completed the training program for the Symani Surgical System; however, the shorter time for the second robotic case represents a clear and significant trend.

**Figure 5.**
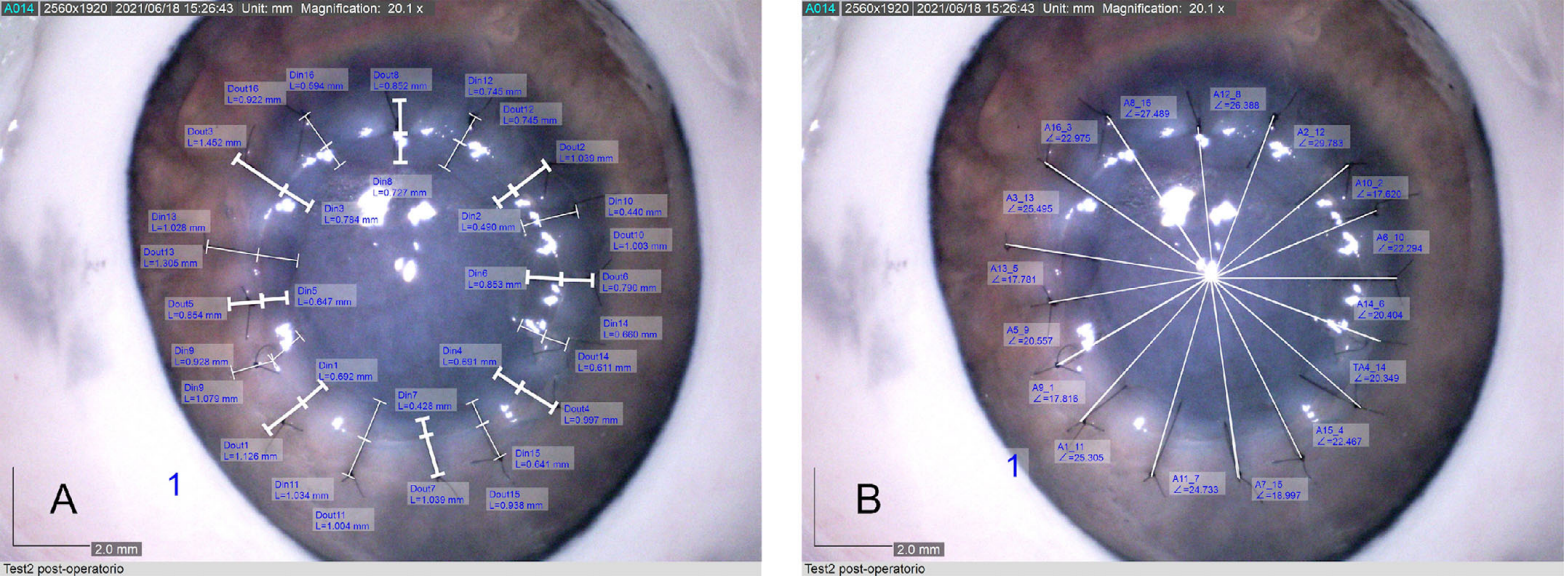
Pictures of measured distances (A) and angles (B) for the 16 interrupted stitches related to the first robotic surgery simulation captured by the Dino Lite digital microscope.

**Figure 6.**
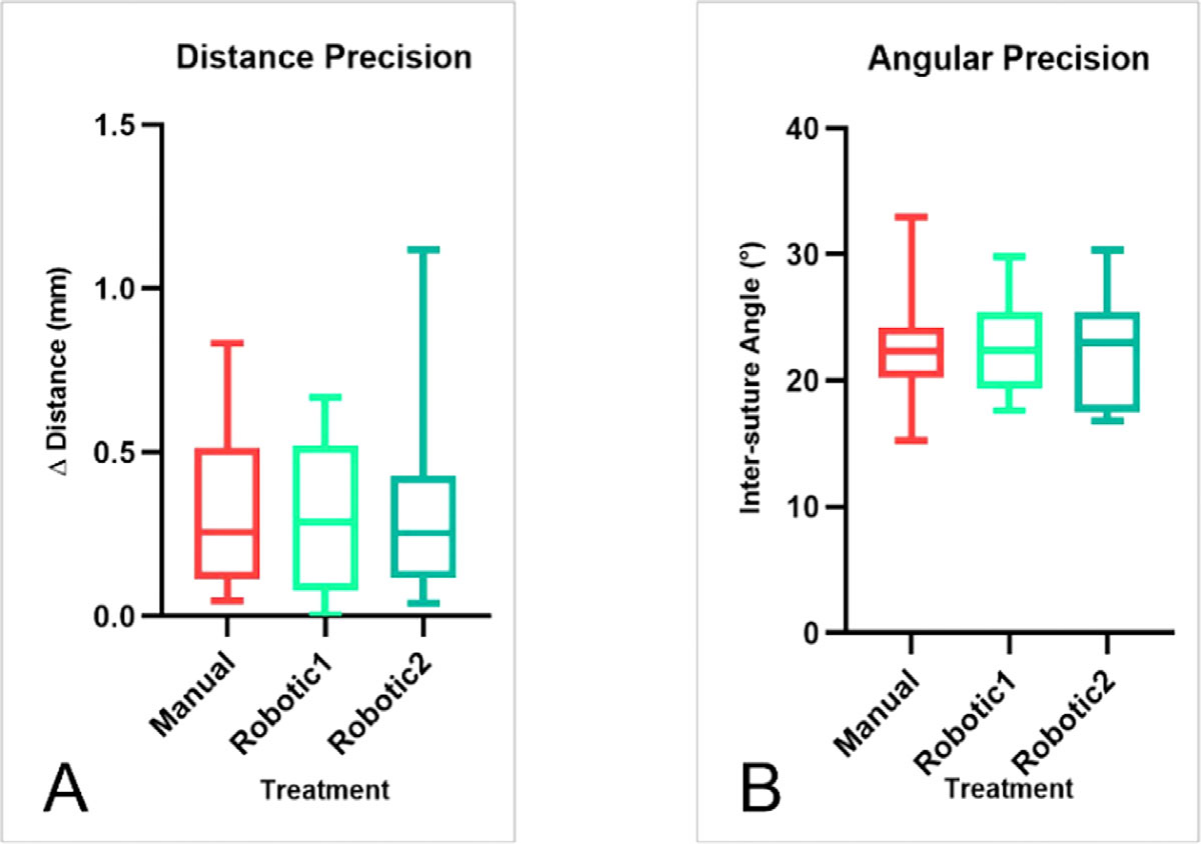
Mean values and value distributions for distance (A) and angular (B) precision measurements.

Images of sutured corneas with measured distances and angles are shown in Figures 5A and 5B, respectively, for the first robotic case. Figures 6A and 6B show the mean values and distributions for the measured samples. The ANOVA test comparing data for both sutures and treatments showed that differences among mean values were not statistically significant (*P* > 0.05), a finding that was associated with both distance and angular precision.

This preliminary result regarding the accuracy of suture placement is promising because it demonstrates an equivalence of robotic versus manual surgery in terms of suturing, even though only two robotic procedures were performed.

Preoperative and postoperative AS-OCT data were used to gain insight about the shape and regularity of the corneal surface.

**Figure 7.**
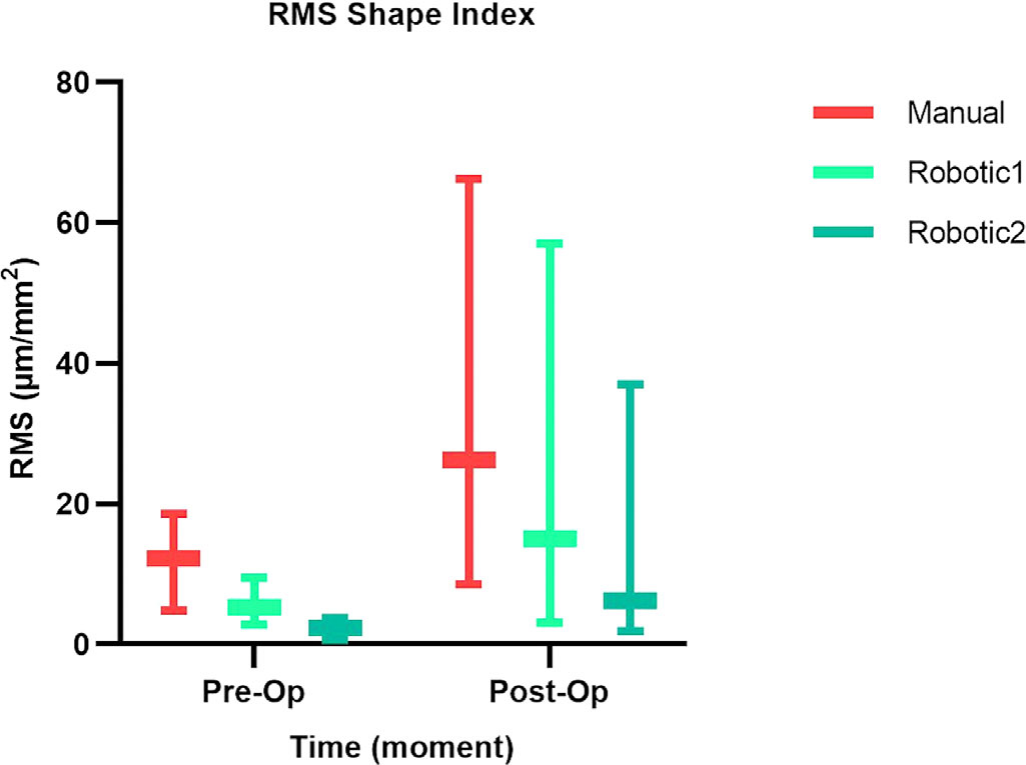
Summary of mean RMS/A values across different corneal radii (3.0, 5.0, and 7.0 mm) for each treatment (manual, robotic1, and robotic2) at two different points in time (preoperatively and postoperatively).

Figure 7 displays summary RMS/A mean values across different corneal radii (3.0, 5.0, and 7.0 mm). Robotic values were generally smaller (i.e., the corneal surface was more regular) than the corresponding manual ones, and the ANOVA test indicated that differences among mean values were not statistically significant (*P* > 0.05) when comparing data for time, treatment, and corneal radius. Again, looking at the surface regularity, for the single surgeon involved and for the three cases examined, the robotic treatment was equivalent to the manual one.

**Figure 8.**
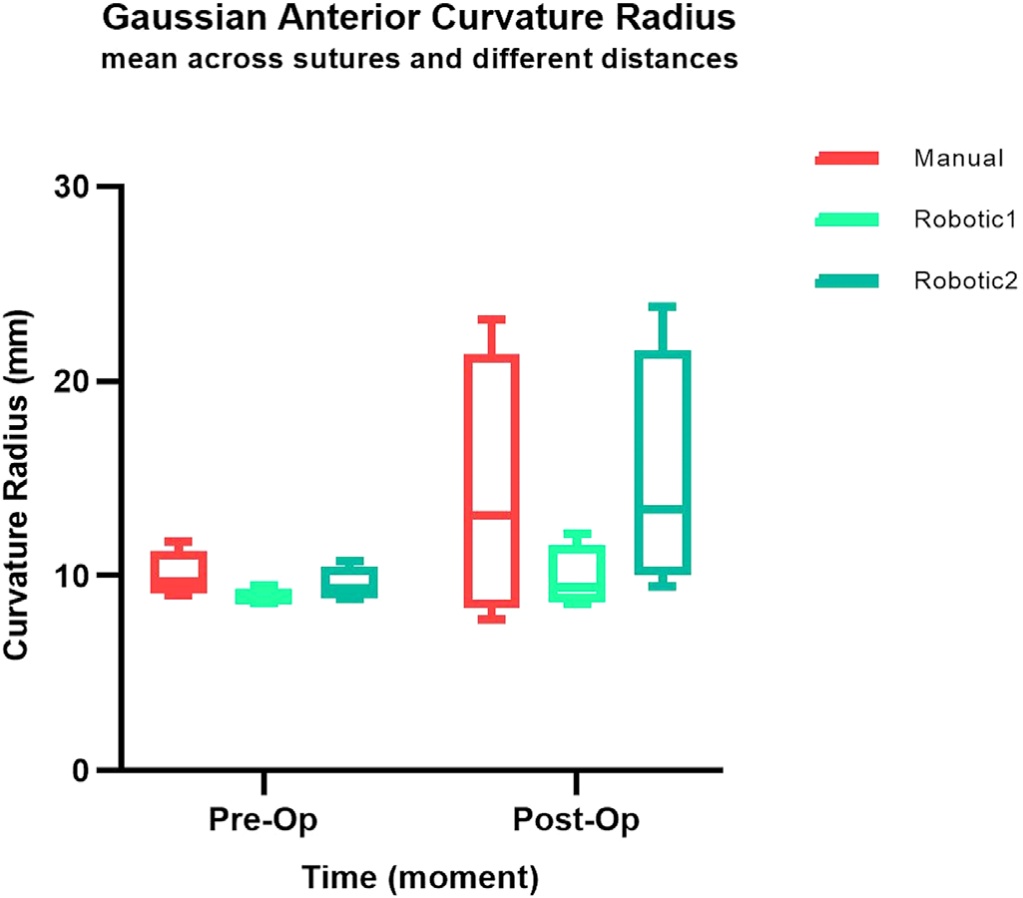
Preoperative versus postoperative mean values of GAC calculated first across sutures and later across different corneal radii.

GAC values extracted for sutured regions were examined in different conditions. Preoperative and postoperative mean values were calculated first across sutures and then across different corneal radii, and they were then compared (Fig. 8). The ANOVA test showed that differences among mean values were statistically significant (*P* < 0.05) when comparing data for time (preoperatively compared to postoperatively), whereas they were not significant when comparing data for treatment and corneal radius.

**Figure 9.**
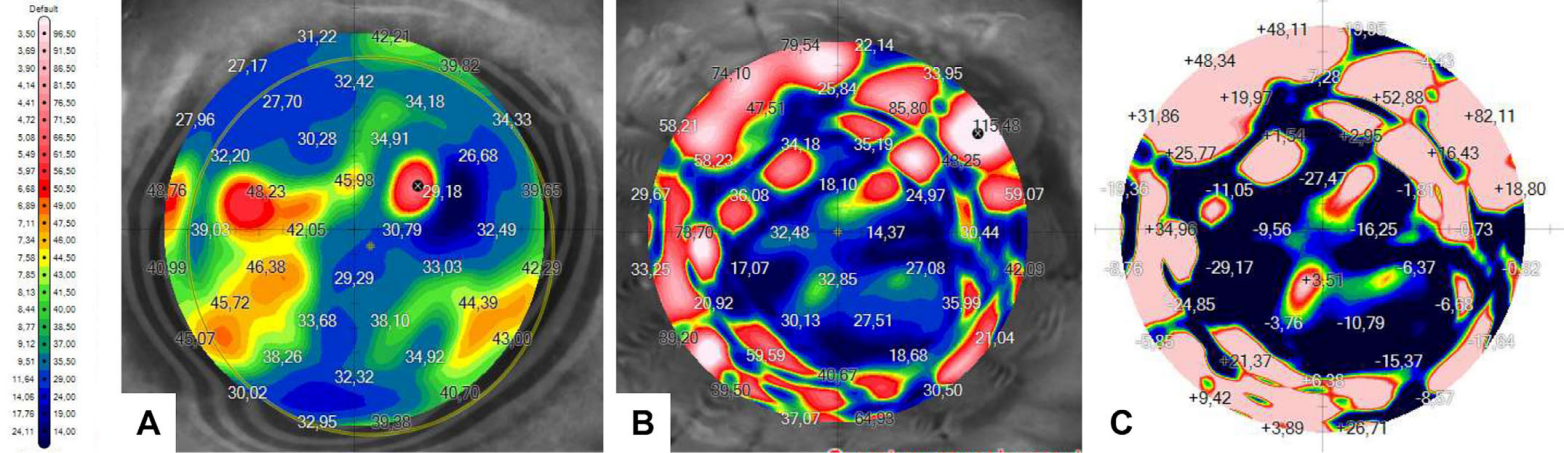
Corneal maps of GAC obtained by the MS-39 for the manual simulation: (A) preoperative, as reference; (B) postoperative; and (C) calculated differences between postoperative and preoperative.

**Figure 10.**
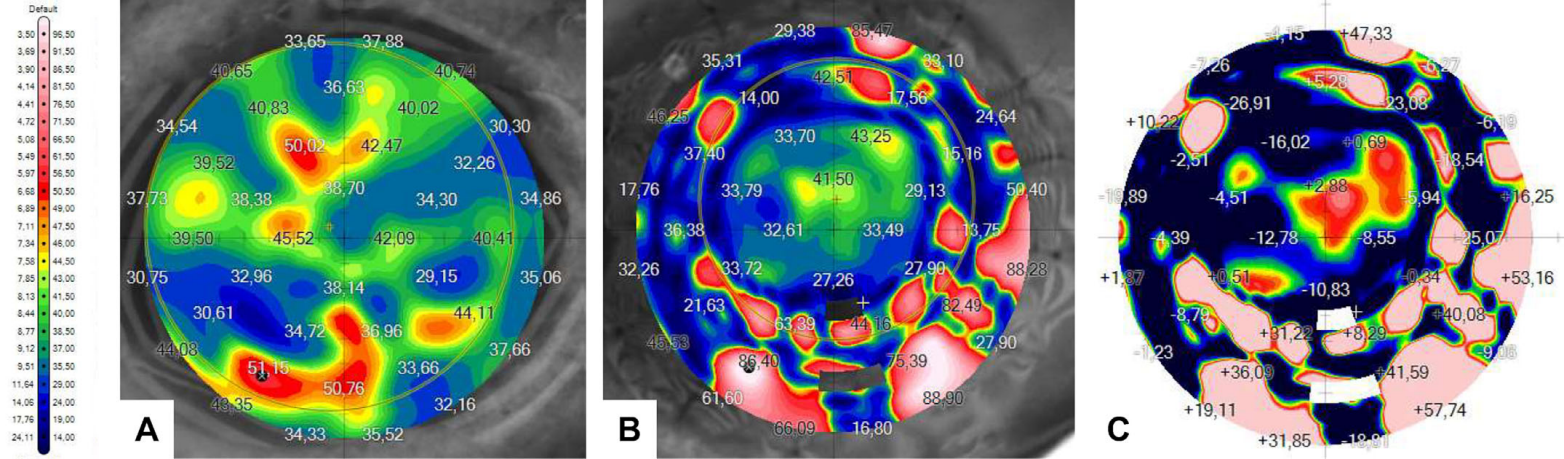
Corneal maps of GAC obtained by the MS-39 for the first robotic simulation: (A) preoperative, as reference; (B) postoperative; and (C) calculated differences between postoperative and preoperative.

**Figure 11.**
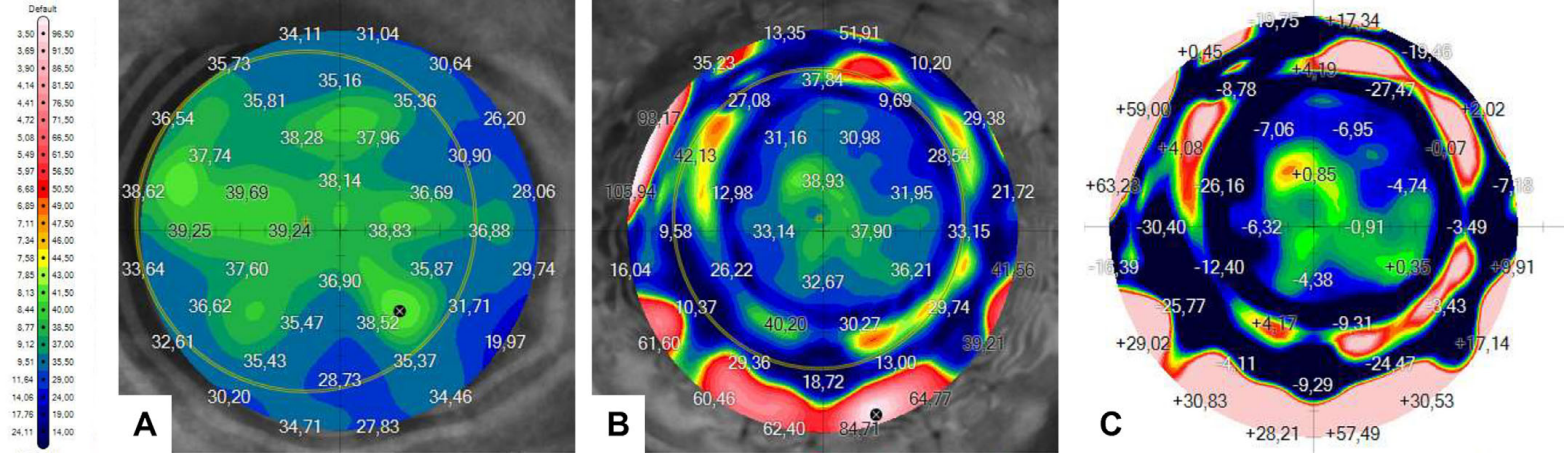
Corneal maps of GAC obtained by the MS-39 for the second robotic simulation: (A) preoperative, as reference; (B) postoperative; and the calculated difference between postoperative and preoperative.

**Figure 12.**
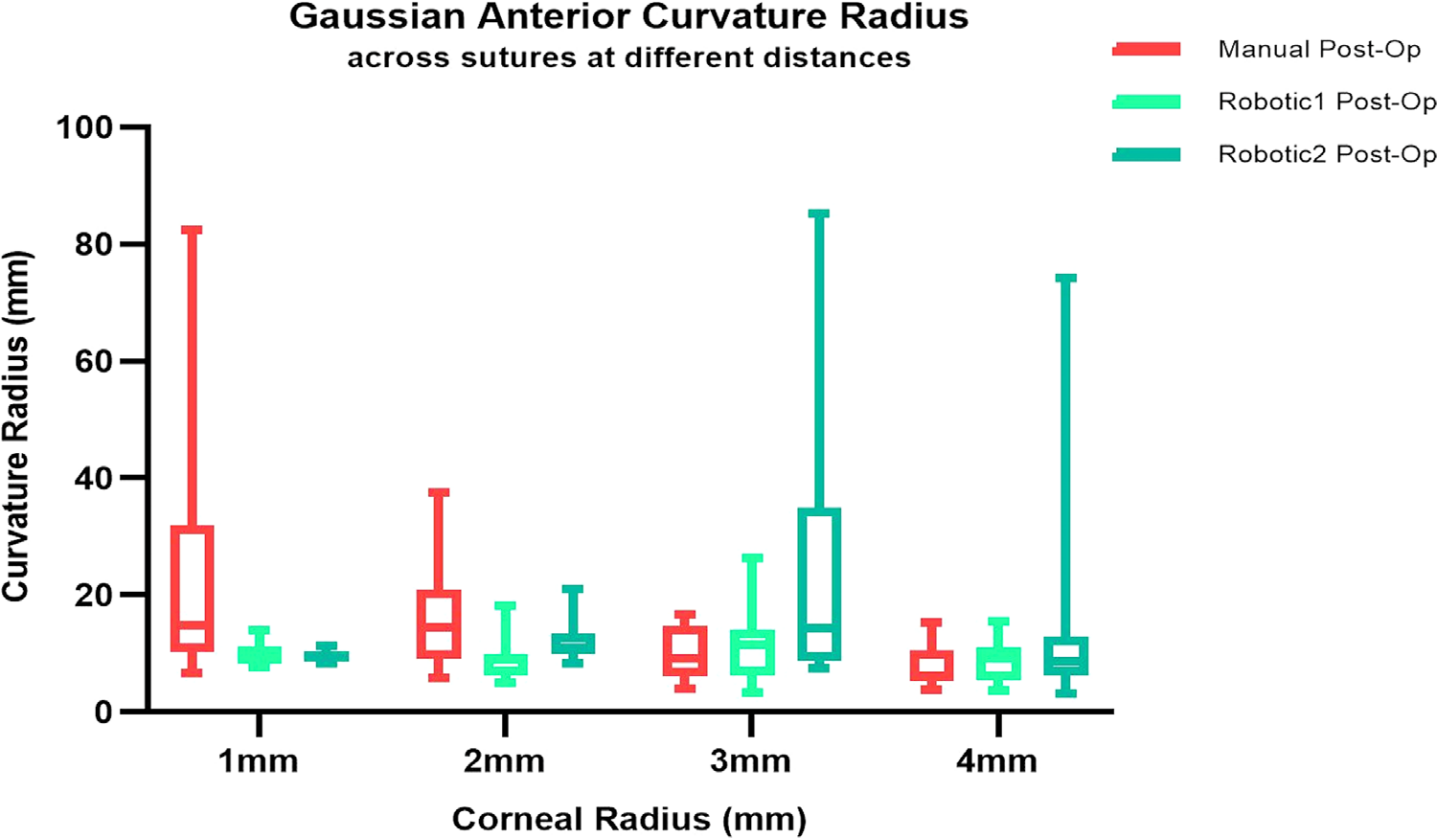
Postoperative GAC values examined across the 16 sutures and plotted against the various corneal radii.

Then, the postoperative data maps were examined across the 16 sutures. Figures 9, 10, and 11 show preoperative and postoperative data and the difference between them obtained by the MS-39 system for all three surgery simulations, respectively. The data were plotted in correspondence with the different corneal radii, as shown in Figure 12. The ANOVA test showed that differences among mean values were statistically significant (*P* < 0.05) when comparing data for treatment (manual vs. robotic), whereas they were not significant when comparing data for sutures and corneal radius. For the three cases considered, there was also a significant interaction between corneal radius and treatment, as the robotic values were reduced with decreasing radius but the manual values improved with increasing radius.

## Discussion

In the ophthalmic surgery field, particularly microsurgery, operations require the surgeon to work using a high-magnification microscope and specialized microsurgical manual instruments. These instruments have tips that are < 1 mm, and sutures are between 0.03 and 0.001 mm (9-0 and 12-0). The introduction of tridimensional magnification devices has led to breakthroughs being achieved in visualizing images, and virtual-reality microscopes have allowed the visualization of high-resolution images that was not possible before. However, little has been done to enhance the surgeons’ capabilities or standardize various surgical techniques. For this reason, we investigated the effectiveness of the Symani Surgical System, which was developed for microsurgical applications and can be used in the ophthalmic field, as well.

The concept of the Symani Surgical System is based on the suggestion that replacing traditional manual practices can lead to certain surgical actions being performed much more smoothly with feedback-controlled motions than could be achieved by the human hand. The main outcomes include the elimination of tissue trauma, superior motion control, and advanced dexterity, and the system requires only a short training time, perhaps shorter than the physical and mental training required today of ophthalmologists. Another important point is that traditional instruments can still be used while taking advantage of robotic systems when needed.

This is the scenario we explored with the Symani Surgical System, as the study aimed to demonstrate its technical feasibility and application to suturing for partial corneal transplant surgery.

Suturing of partial corneal graft with the robot was successfully performed in the ex vivo porcine model, as confirmed by data analysis results. Moreover, the combination of the robotic system with a 3D microscope provided both good visualization and operative setup, as well as controlled and appropriate placement of corneal sutures in the corneal wound.

Although the porcine eye is a valid model for clinical practice training in ophthalmic applications, the porcine cornea differs significantly from the human cornea in terms of thickness. In particular, the peripheral zone is thicker than in the human cornea, which reaches an average maximum value of about 650 µm under healthy conditions.^14^ Considering the corneal thickness of the selected model and the use of a trephine commonly used in standard clinical practice, which allows for a full trephination of a maximum of 800 µm, for this feasibility study the trephination proved to be partial. This resulted in not evaluating some typical intraoperative variables; however, in this feasibility study we were focused on confirming the applicability of a dedicated microsurgical robot for the ophthalmic field.

The choice to utilize the interrupted stitches technique, which is commonly used in clinical practice, allowed us, on the one hand, to test a robotic instrument with double functionality (needle holder and suture cut), and, on the other, to obtain additional intraoperative suturing data to compare robotic performance to manual. In particular, application of the interrupted stitches technique allowed also to collect more intraoperative data regarding use of the Symani Surgical System and to perform statistical analyses. During each surgery simulation, the various steps of each single stitch were timed, which allowed data analysis based on averaged parameters over 16 points for each procedure (16 points for the manual simulation and 32 total for the robot simulations).

The presented results do not have any statistical significance due to the small sample size. Only three surgical treatments (one manual as reference and two robotic) were performed by the same surgeon, but the technique shows promise, as results of the robotic suturing treatments and the manual treatment were very similar. This equivalence was found for distance and angular precision, as well as for surface regularity described by the RMS index.

With regard to total suturing time, including cutting time, both of the robotic suturing execution times were longer than the manual procedure, but the second robotic simulation was 20 minutes shorter than the first one, demonstrating a trend toward approaching the time required for manual execution. It is important to keep in mind that the surgeon had completed only 4 hours of training with the Symani Surgical System before the feasibility simulations were performed. Our results are confirmed by the literature, including studies by van Mulken et al.^16^ and Ballestín et al.^17^ The robotic-assisted surgery proved to require longer surgical times compared with the manual microsurgery technique, but practice with the technique will allow microsurgeons to improve their performance. Although the surgeon did not complete the entire training program for the robotic technique, he rapidly became confident and proficient with its use. It is important to mention that, in clinical practice, stitch completion time is not a key clinical outcome for suture success. All of these results support and demonstrate the technical feasibility, at least in the porcine model, of robotic suturing in corneal transplant using the Symani Surgical System and warrant consideration for experimental studies with greater sample sizes and evaluation in controlled human trials.

With regard to the near future, robotic-assisted surgery has the potential to be used for telesurgery, where surgeons might be physically located many miles away from where their expertise is needed to provide training or patient care. In this case, the ideal system might be a computer-assisted robot rather than a fully automated system.

## Supplementary Material

Supplement 1

## Supplementary Material

Supplementary Movie S1. Recording of single suture robotically performed by a surgeon using the Symani Surgical System with 10× scaling factor.
